# Enzyme mediated nanofibrillation of cellulose by the synergistic actions of an endoglucanase, lytic polysaccharide monooxygenase (LPMO) and xylanase

**DOI:** 10.1038/s41598-018-21016-6

**Published:** 2018-02-16

**Authors:** Jinguang Hu, Dong Tian, Scott Renneckar, Jack N. Saddler

**Affiliations:** 1 Department of Wood Science, Forest Products Biotechnology/Bioenergy Group, Faculty of Forestry, University of British Columbia, 2424 Main Mall, Vancouver, British Columbia V6T 1Z4 Canada; 2 Department of Wood Science, Advanced Renewable Materials Lab, Faculty of Forestry, University of British Columbia, 2424 Main Mall, Vancouver, British Columbia V6T 1Z4 Canada; 3 Institute of Ecological and Environmental Sciences, Sichuan Agricultural University, Chengdu, Sichuan 611130 PR China

## Abstract

Physiochemical methods have generally been used to “open-up” biomass substrates/pulps and have been the main method used to fibrillate cellulose. However, recent work has shown that canonical cellulase enzymes such as endoglucanases, in combination with “amorphogenesis inducing” proteins such as lytic polysaccharide monooxygenases (LPMO), swollenin and hemicellulases, are able to increase cellulose accessibility. In the work reported here different combinations of endoglucanase, LPMO and xylanase were applied to Kraft pulps to assess their potential to induce fibrillation at low enzyme loading over a short time period. Although gross fiber properties (fiber length, width and morphology) were relatively unchanged, over a short period of time, the intrinsic physicochemical characteristics of the pulp fibers (e.g. cellulose accessibility/DP/crystallinity/charge) were positively enhanced by the synergistic cooperation of the enzymes. LPMO addition resulted in the oxidative cleavage of the pulps, increasing the negative charge (~100 mmol kg^−1^) on the cellulose fibers. This improved cellulose nanofibrilliation while stabilizing the nanofibril suspension (zeta potential ζ = ~60 mV), without sacrificing nanocellulose thermostability. The combination of endoglucanase, LPMO and xylanases was shown to facilitate nanofibrillation, potentially reducing the need for mechanical refining while resulting in a pulp with a more uniform nanofibril composition.

## Introduction

To improve their profitability, many forest products companies are in the process of diversifying their product portfolios to produce new, higher-value/higher growth products for use in non-traditional pulp markets. One promising new product is nanofibrillated cellulose (NFC) which has potential applications in areas such as polymer composites and energy storage devices^1,2^. Due to its superior mechanical properties and large surface area, NFC can be used in applications such as rheology modifiers, hydrogels, supercapacitors, biosensors and flexible transparent displays^3,4^. The most commonly used method to make NFC is via mechanical refining utilizing equipment such as homogenizers, micro-fluidizers and grinders. However, cost-effective production of NFC’s by these physicochemical approaches remains challenging due to the high energy requirements of refining and the heterogeneity of the material obtained after refining^5,6^.

Previous attempts to reduce refining energy, while improving product homogeneity, have mainly focused on the use of various cellulose chemical derivatization pretreatment processes such as TEMPO-oxidation, carboxymethylation and cationization^7–11^. However, despite some initial success, these chemical processes have proven to be relatively expensive. There have also been some concerns raised about potential environmental risks^2^, which might limit their further industrial expansion. In contrast, biological mediated pretreatments can be carried out in milder, aqueous environments. This approach has received increasing attention^12^, partially due to the high substrate specificity of enzymes^13^.

As well as the potential role that more “classical” cellulases such as endoglucanase and exoglucanase have been shown to play in facilitating refining and fibrillation^12,14–19^, more recent work has also shown how “accessory” enzymes/proteins such as xylanases, swollenin, expansin-like proteins and lytic polysaccharide monooxygenases (LPMO) can boost the hydrolytic performance of cellulase cocktails, primarily by improving accessibility of the enzymes to the cellulosic component^20–23^. Although these accessory enzymes do not directly hydrolyze cellulose, they have been shown to have high specificity in selectively modifying the carbohydrate network in a relatively fast manner^21,22,24^. For example, xylanases have been shown to both selectively remove surface xylan and act synergistically with cellulases to enhance fiber characteristics by increasing fiber swelling and porosity^24^. It has also been shown that the oxidative cleavage of the accessible crystalline cellulose region by LPMO’s not only disrupts and opens-up the highly organized cellulose structure, it also results in the deposition of carboxylic acid groups on the fiber surface^25–27^. The resulting, enhanced, negative charge on the cellulose fibers should prove beneficial to cellulose fibrillation due to an increase in repulsive force and reduced fiber aggregation, (in the same way as TEMPO oxidation has been shown to enhance fibrillation)^7^. Although recent work has shown how LPMO treatment can enhance cellulose nanofibrillation^28^, the potential of cocktails of “accessory” enzymes/proteins to further enhance NFC production and properties has not yet been assessed.

In the work reported here we assessed the potential of individual and combinations of an endoglucanase, LPMO auxiliary family 9 (AA9) and endoxylanase to enhance pulp fibrillation and properties. Potential changes to major cellulose fiber physicochemical characteristics such as carbohydrate content, gross fiber morphology/accessibility, cellulose degree of polymerization and crystallinity were assessed after enzymatic treatment. The work reported below indicated that the enzyme combination of endoglucanase, AA9 and endoxylanase could “open-up” and “loosen” the fiber structure quickly and effectively, with minimum carbohydrate loss, while facilitating cellulose nanofibrillation. The results suggest that enzyme mediated treatments have the potential to reduced refining energy requirements while resulting in more uniform nanofibrils.

## Results and Discussion

A fully bleached Kraft pulp (BKP) composed of 77% glucan, 17% xylan and less than 1% lignin was selected as the initial feedstock to assess the potential of individual and combinations of endoglucanase (Novozyme 476), lytic polysaccharide monooxygenase (LPMO) auxiliary activity family 9 (AA9) and endoxylanase to facilitate cellulose nanofibrillation over a range of pH’s (data not shown). Although AA9 and endoxylanase exhibited highest enzyme activities at pH 5, as the endoglucanase activity of Novozymes 476 has been reported to be effective at both pH 5.0 and pH 7.0 when it was used in the pulp and paper appications^12,19,29^, the reduction in cellulose degree of polymerization (DP), (a common indicator for endoglucanase activity), was initially assessed at both pH 5.0 and 7.0. As it was apparent that endoglucanase activity was greatest at pH 5.0, regardless of the enzyme loading used (Fig. S1), all subsequent enzyme treatment were carried out at pH 5.0.

**Figure 1:**
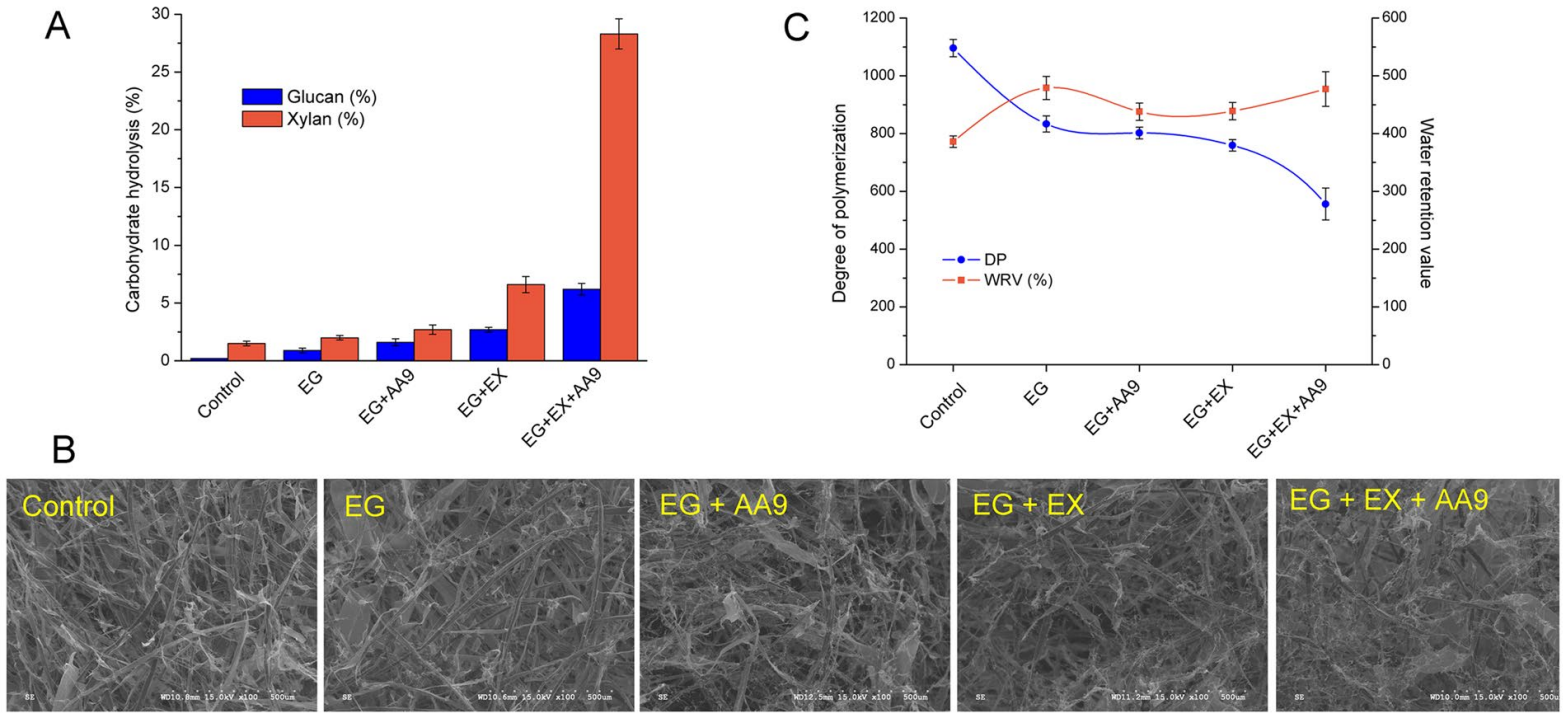
(**A**) Enzyme mediated hydrolysis of the cellulose and xylan component of Bleached Kraft Pulp (BKP). (**B**) Scanning electric microscopy (SEM) images (100×) of Bleached Kraft Pulp (BKP) after various enzyme mediated treatments. (**C**) The degree of polymerization (DP) and water retention values (WRV) of enzyme treated pulp. EG: endoglucanase; AA9: lytic polysaccharide monooxygenase auxiliary activity family 9 enzyme; EX: endoxylanase.

Earlier work had indicated that enzyme treatments could be effective at relatively low protein loadings within a short period of time^12,19^. Thus, an enzyme loading of 1 mg enzyme per g cellulose for 3 hours was initially assessed, as we wanted to maximize beneficial changes while minimizing any possible deconstruction of the fiber. It was apparent (Fig. 1A) that the addition of individual and binary combinations of enzymes resulted in negligible pulp hydrolysis although the ternary mixture of endoglucanase, endoxylanase and AA9 resulted in significant xylan (30%) and cellulose (7%) hydrolysis. When the fiber morphology of the enzyme treated pulps were assessed by Fiber Quality Analysis (FQA) the gross fiber length and width, with and without enzyme treatments, were comparable (around 750 μm and 21 μm for fiber length and width, respectively, as shown in Table S1). In contrast, scanning electron microscopy (SEM) (Fig. 1B) indicated that the surface morphology of the various fibers was disrupted to varying extents by the different enzyme combination. The most significant disruption occurred when the ternary enzyme mixture of endoglucanase, endoxylanase and AA9 was used.

To complement the FQA and SEM analyses, which indicated the gross morphology of the fibers, the degree of polymerization (DP), crystallinity (CrI) and water retention values (WRV) of the enzyme treated pulps were also assessed, to see if these values could help provide further insights into the enzyme induced changes to the physical characteristics of the fibers. Generally, the endoglucanase treatment on its own resulted in a DP reduction of approximately 20% (from ~1096 to ~833) (Fig. 1C) and a CrI increase of 10% (from ~53% to ~61%) (Fig. S2), while the addition of the endoxylanase and/or AA9 to the endoglucanase further intensified the observed changes (Figs 1C and S2). It was also apparent that, after each of the enzyme treatments, the water retention values (WRV), which can be considered to be an indicator of overall fiber accessibility, showed a contrary trend to what was observed with changes to the DP (Fig. 1C).

**Figure 2:**
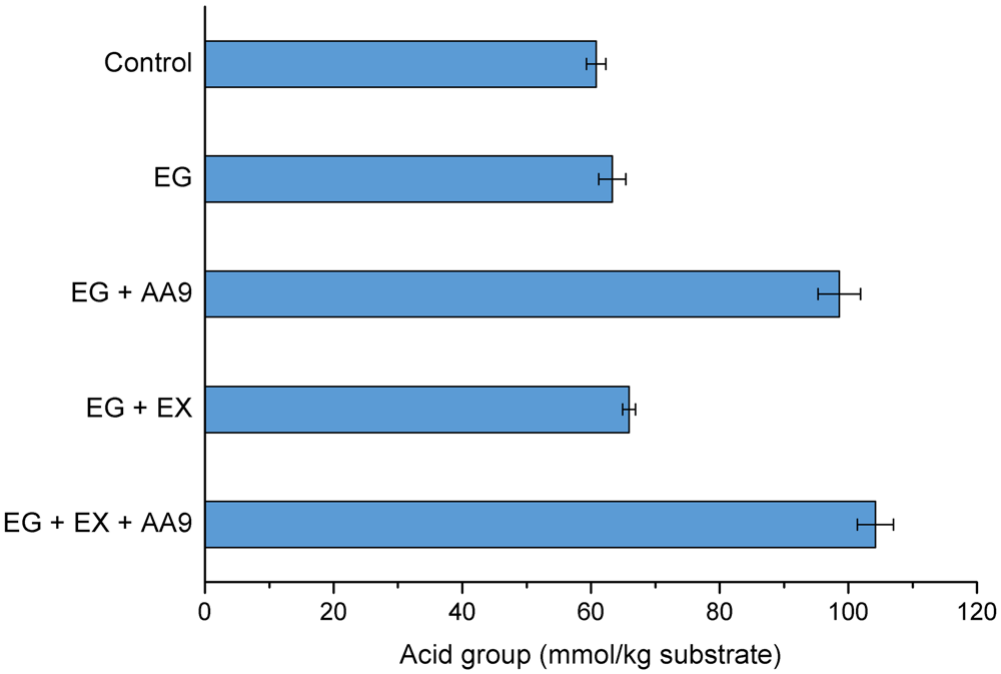
Acid group content of Bleached Kraft Pulp (BKP) after treatment by various enzyme combinations. EG: endoglucanase; AA9: lytic polysaccharide monooxygenase auxiliary activity family 9 enzyme; EX: endoxylanase.

**Figure 3:**
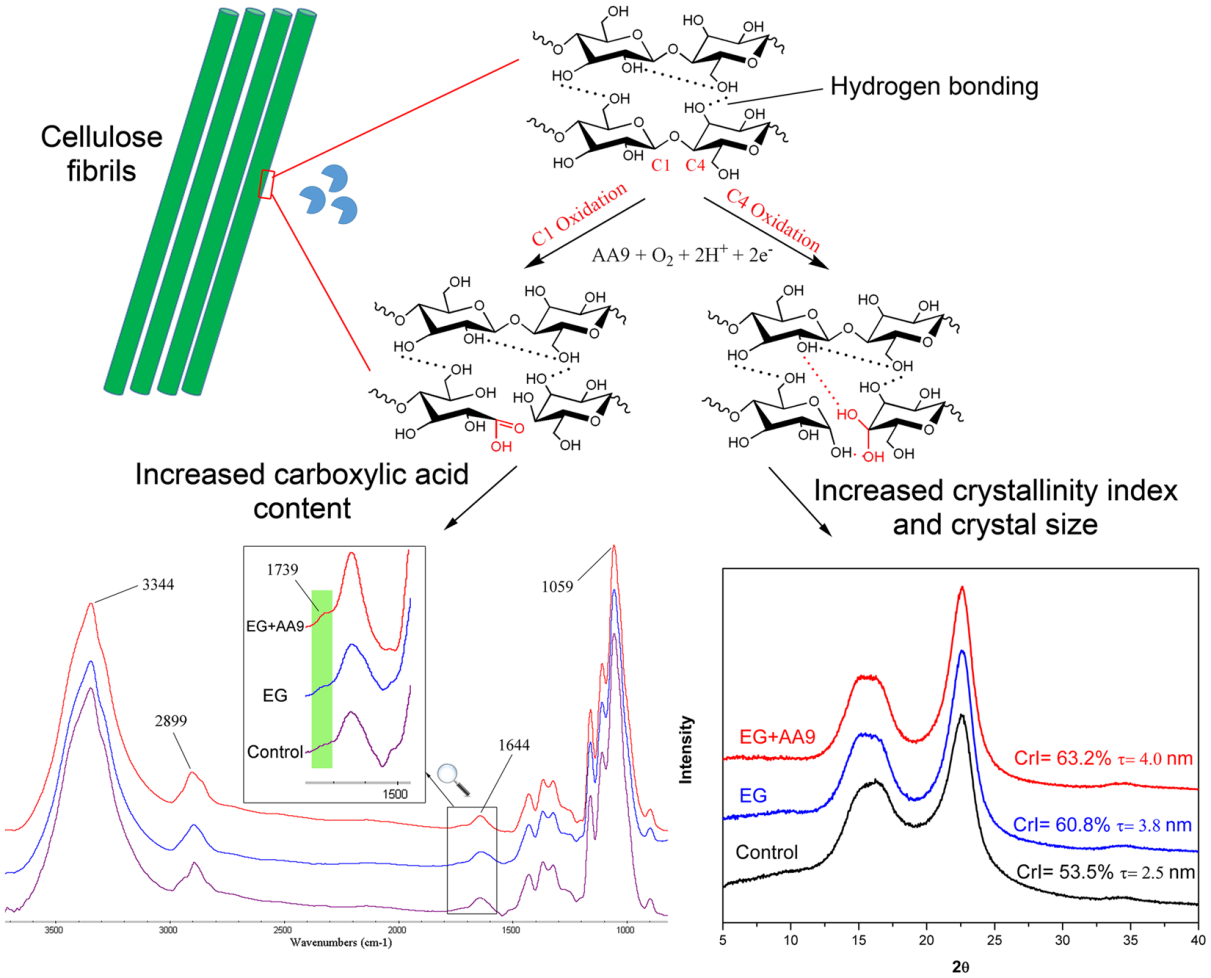
Diagrammatic representation of the potential mechanisms behind AA9 induced fiber modification. The C1 or C4 oxidation of cellulose via AA9 oxidative cleavage introduces carboxyl groups and/or enhances the hydrogen bonding network of the cellulose fiber, respectively. EG: endoglucanase; AA9: lytic polysaccharide monooxygenase auxiliary activity family 9 enzyme.

As well as assessing possible changes to the major fiber physical characteristics after the various enzyme treatments, FTIR was also used to indicate if there were corresponding changes to the molecular structure of the pulp (Fig. S3). It appeared that the various enzyme treatments resulted in minimal changes as the typical absorption bands associated with cellulose remained unchanged (3344 cm^−1^ for –OH stretching, 1059 cm^−1^ for –C–O– stretching vibration in glucose ring) (Fig. S3). However a weak signal associated with carboxyl groups (at 1739 cm^−1^) was detected when AA9 was included in the enzyme treatment. This was anticipated as the oxidative cleavage of cellulose by AA9 results in the oxidation of either the C_1_ or C_4_ carbon, producing a carboxylic acid or ketone structure, respectively. This was similar to previous observations where FTIR analysis showed that TEMPO treated cellulose resulted in a reduction in the DP of the fibers while introducing carboxylic acid groups to the C_6_^30^. Further quantification of the carbonyl groups using conductometric titration indicated a more than 50% increase in the carboxylic acid group content (from ~60 to ~100 mmol kg^−1^) of the fibers after AA9 treatment (Fig. 2). Although the increased carboxylic acid content was a good indicator that enhanced cellulose nanofibrillation had occurred, it proved problematic in trying to rationalize the observed increase in cellulose CrI after AA9 treatment (Fig. S2). It has been shown^26,31^ that AA9 treatment resulted in production of equivalent amount of ketone structures from C_4_ oxidation, as well as carboxylic acid groups from C_1_ oxidation. However, the carbonyl groups on the ketone structure after C_4_ oxidation were shown to subsequently convert to 4-ketoaldoses (gemdiol) groups^28^, further enhancing the intermolecular hydrogen bonding network and, consequently, the crystallinity of the cellulose fibers (Fig. 3). The likelihood that this was indeed occurring was further supported by the fact that the addition of small amounts of Cel6A (a non-reducing end attacking exoglucanase that can hydrolyze the C_4_ oxidized ketone structure) to the enzyme cocktail eliminated the increase in the CrI of the cellulose when combined with AA9 (Fig. S2).

**Figure 4:**
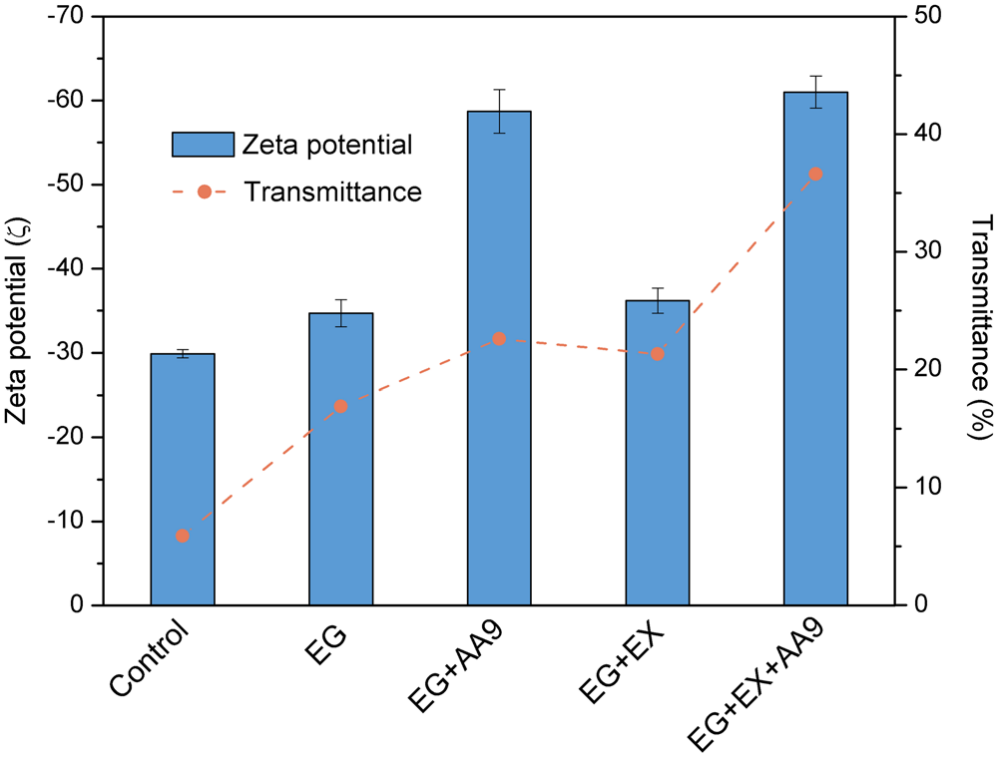
The zeta potential (ζ) and optical transmittance (at 600 nm) of Bleached Kraft Pulp suspensions (0.1%, w/v) treated with various enzyme combinations. EG: endoglucanase; AA9: lytic polysaccharide monooxygenase auxiliary activity family 9 enzyme; EX: endoxylanase.

We next assessed the possible benefits of applying sonication after each of the enzyme treatments to enhance nanofibrillation where the zeta potential (ζ) values and visible light transmittance (at 600 nm) were used respectively to assess suspension stability and the extent of cellulose nanofibrillation. It was apparent that enzyme treatment greatly improved both the zeta potential and fiber suspensions values (Fig. 4), indicating the benefits of sonication in enhancing cellulose nanofibrillation. Although endoglucanase treatment on its own slightly increased the ζ value of the bleached fiber from ~30 to ~35 mV), the AA9 treatment resulted in the greatest increase in ζ values (~60 mV, Fig. 4). It was likely that the increase in the ζ value was due to the increase in carboxyl groups resulting from AA9 oxidative cleavage (Figs 2 and S3). Since a ζ value greater than ±40 mV is usually required to result in a suspension solely stabilized by electrostatic repulsion, it was also likely that the improved ζ value resulting from AA9 treatment helped self-stabilize the nanocellulose suspension. It was apparent that enzyme treatments improved the visible light transmittance of the sonicated fiber suspension (Fig. 4), with endoglucanase treatment doubling the percentage of light transmittance of the fiber suspension. Although the synergistic cooperation between endoglucanase and either AA9 or endoxylanase further increased transmittance level, the most significant improvement resulted from the ternary combination of all three enzymes (Fig. 4), as an up to 7 times (35%) increase in transmittance levels was observed as compared to the control sample (5%).

**Figure 5:**
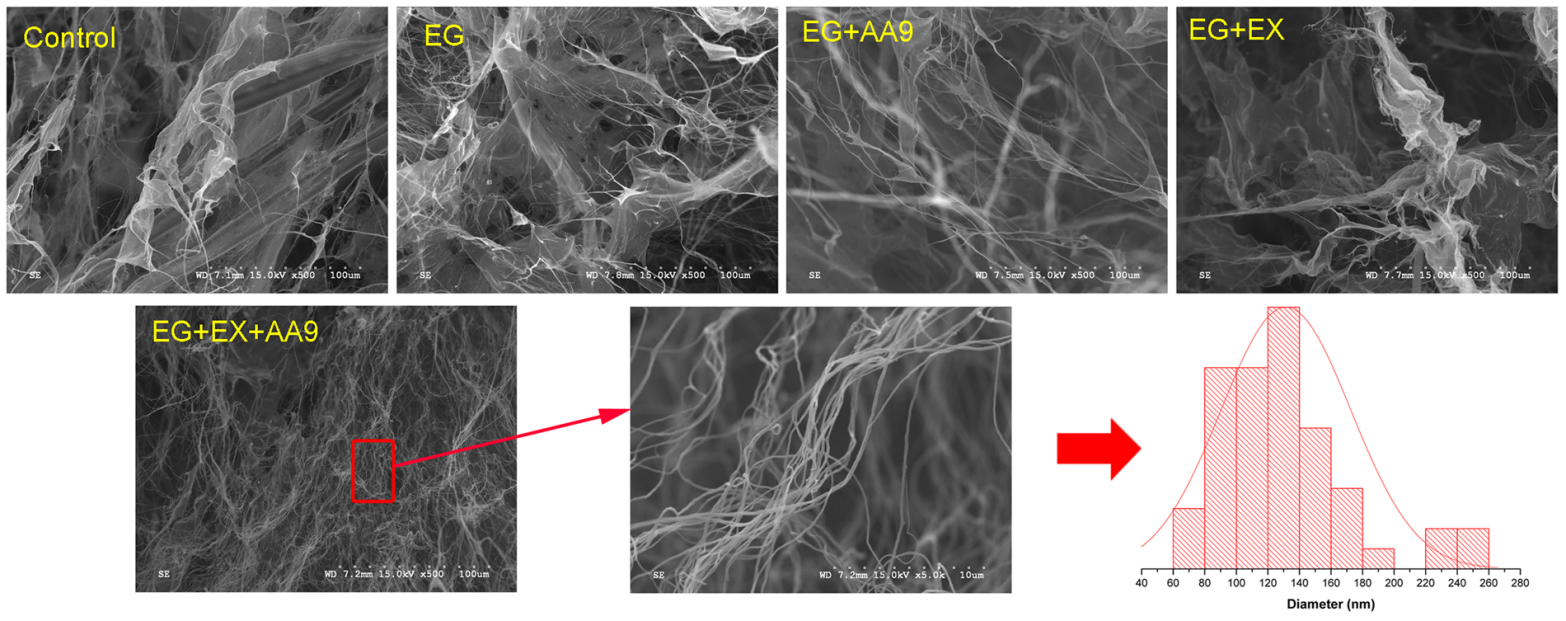
SEM images (**A**) of an enzyme treated Bleached Kraft Pulp (BKP) fiber suspension after sonication, (**B**) Fiber width distribution of EG + EX + AA9 treated pulp. EG: endoglucanase; AA9: lytic polysaccharide monooxygenase auxiliary activity family 9 enzyme; EX: endoxylanase.

The extent of cellulose nanofibrillation of the sonicated fiber suspensions was next assessed by direct visualization of the lyophilized samples, using SEM. Unlike traditional mechanical refining methods (e.g. high-pressure homogenization and microfluidization) which directly disintegrate pulp fibers to nanofibrillated cellulose, it appeared that the milder sonication process was less able to disrupt the original pulp fiber structure (Fig. 5A). However, endoglucanase treatment alone or in combination with either AA9 or endoxylanase seemed to facilitate cellulose fibrillation to some degree, as a highly entangled nano and/or micro fiber network was observed (Fig. 5B–D). It appeared that synergistic cooperation between endoglucanase, AA9 and endoxylanase enhanced fiber disintegration with non-aggregated, individual, cellulose nanofibrils clearly visible (Fig. 5E). These observations were in agreement with previous visible light transmittance analysis which had indicated these structures were smaller than the wavelength of light (Fig. 4). When image J software was used to measure the cellulose nanofibrils (Fig. 5E) the nanoscale fibrillated cellulose had an average width of 130 nm and a distribution from 60 to 260 nm (Fig. 5B).

It is widely recognized that the thermostability of nanocellulose is an important parameter that significantly influences its potential in applications such as polymer composites. As earlier work^8^ had shown that many of the chemical pretreatment processes used to enhance cellulose fibrillation reduced the thermostability of the resulting fibers, this characteristic was next assessed. It was apparent, using thermogravimetric analysis (TGA), that endoglucanase treatment reduced both the initial decomposing temperature (T_i_) and the temperature at the maximum decomposing rate (T_max_) of the final fiber suspension (Table 1 and Fig. S4). While AA9 and xylanase treatments on their own had no effect of fiber thermostability (data not shown). Surprisingly, AA9 treatment enhanced the T_i_ and T_max_ of the fiber suspensions back to their original levels (control sample without enzyme treatment). Although previous work has shown that chemical based TEMPO-oxidation pretreatment greatly improved the efficacy of cellulose nanofibrillation, the large amount of carboxylic acid groups introduced onto the C6 primary hydroxyls of cellulose significantly decreased the thermostability of the nanofibrillated cellulose^32^. As oxidative cleavage mediated by the AA9 enzyme has been shown to introduce some carboxyl groups onto the cellulose (Figs 2 and S3) and it was initially anticipated that AA9 might also decrease the thermostability of cellulose fiber. However, it appeared that the slight increase in carboxyl groups via C1 oxidation had only a limited influence on fiber thermostability. Thus, it was likely that the enhanced crystallinity (Figs 3 and S2) had a greater influence on fiber thermostability.

**Table 1:** The thermostability of enzymatically pretreated bleached kraft pulp (BKP) fiber after sonication process.

Sample	Ti (°C)	Tmax (°C)
Control	303.4 ± 1.3	346.9 ± 0.5
EG	289.8 ± 0.5	340.4 ± 0.8
EG + AA9	300.5 ± 2.1	347.7 ± 1.9
EG + EX	293.5 ± 0.9	341.9 ± 1.8
EG + EX + AA9	302.4 ± 1.1	348.3 ± 2.2

EG: endoglucanase; AA9: lytic polysaccharide monooxygenase auxiliary activity family 9 enzyme; EX: endoxylanase. The experiment were performed in triplicate and the mean values and errors bars were calculated. The statistical analysis showed that the difference were statistically significant (one way ANOVA, p < 0.05).

Although previous work has shown that endoglucanase treatment can aid in the fibrillation of nanocellulose via fiber embrittling/shortening/softening^12,19^, in the work reported here, it was also apparent that endoglucanase treatment, in combination sonication, resulted in an even more fibrillated structure (Fig. 5). However, additional mechanical energy will likely be required to fully fibrillate the samples. As the bleached Kraft pulp has a significant amount of xylan associated with the fiber and the xylan has been shown to coat the surface of the microfibrils^24,33^, it was probable that xylanase treatment enhanced endoglucanase accessibility to the cellulose while also reducing intermicrofibril bonding. Although AA9 treatment resulted in near equivalent acid groups on the cellulose pulps, with or without xylanase treatments, the DP of the cellulose was significantly reduced after xylanase treatments. This suggested that, to some extent, both the AA9 and endoglucanases cleaved the cellulose chain after xylanase treatment. These results suggested that the role that xylan plays in fibrillation deserves further attention as, in some cases, the retention of xylans is critical such as reducing hornification^34^. When the fibrillation of the various enzyme pretreated fibers was compared (Fig. S5), it appeared that enzyme treatments with the endoglucanases, AA9 and xylanases combination resulted in more uniformly dispersed nanofibers with smaller diameters (Fig. 5). In summary, the work reported here has shown that the synergistic action of endoglucanases, AA9, xylanases and sonication enhanced the fibrillation and production of nanofibrillated cellulose derived from a Kraft pulp.

## Conclusions

Selective enzyme treatments combined with sonication facilitated the mechanical disintegration of a bleached Kraft pulp to nanofibrillated cellulose. The resulting very low energy input required to produce nanofibrillated cellulose was probably due to an increase overall substrate accessibility resulting from the synergistic action of the endoglucanase, AA9 and xylanase on the Kraft pulp. It was apparent that the hemicellulose component of the bleached Kraft pulp played a significant role in hampering the separation of cellulose nanofibrils. The low enzyme loadings and short incubation times required indicate the considerable potential that selective enzyme additions have in the production of higher value nanofibrillated or nanocrystalline cellulose products.

## Methods

### Materials

The starting substrate used in this study is hardwood Kraft pulp, which was bleached twice to remove lignin before further use. The major chemical components of the bleached Kraft pulp (BKP) were analyzed according to TAPPI Standard Method T-222: glucan 77.1%, xylan 17.3%, lignin <0.5%. Briefly, approximately 0.2 g oven dry weight of the ground sample (40-mesh) was added to 3 mL of 72% sulfuric acid. The sample was stirred every 10 minutes for 2 h before being diluted to 4% acid concentration by the addition of 112 ml of water. The diluted samples were subsequently transferred to an autoclave set at 121 °C for 1 hour for a second stage of acid hydrolysis. After cooling, the acid hydrolyzed samples were vacuum filtered through oven dried (105 °C) and pre-weighed sintered glass crucibles (medium coarseness). The carbohydrate composition of acid hydrolysate in filtrate was determined by high performance anion exchange chromatography (Dionex DX-3000, Sunnyvale, CA). Acid soluble lignin was determined by reading the absorbance at 205 nm on a Cary 50 UV-Vis spectrometer. The acid insoluble lignin recovered in the crucibles was determined gravimetrically by drying the insoluble lignin in an oven overnight.

BKP was first soaked in distilled water overnight and then solvent-exchanged into acetate buffer (pH 5.0) by vacuum filtration. To obtain well dispersed suspension of BKP in the acetate buffer, BKP was disintegrated by a lab blender (Waring, Canada) for 20 min. Endoglucanase, *Thermoascus aurantiacus* AA9, are kindly provided by Novozymes. Exoglucanase and xylanase were purified from commercial enzyme preparation Celluclast and HTec, respectively, according to^20,35^.

### Enzymatic pretreatment and nanofibrillation of BKP

BKP was enzymatically treated by endoglucanase and other accessory enzymes at an enzyme loading of 1 mg of each enzyme per g substrate (dry weight). The reaction was conducted in 50 mM acetate buffer at pH 5.0 with a solid concentration of 1% (w/v, weigh per volume), incubated at 50 °C for 3 h (Combi H12, FINEPCR Co., Ltd. Korea). In the case of AA9 supplementation, the reducing cofactor gallate, at a concentration of 10 mM, was added to the reaction system to promote AA9 activity^21,23^. The control experiments showed that the addition of gallate at this concentration had no effects on the pulp properties. Protein concentration was quantified by Ninhydrin-based assay35. After enzymatic pretreatment, the enzyme cocktail was denatured by placing in the water bath at 95 °C for 15 min and then the liquid fraction was collected for released sugars analysis. The resultant BKP was washed with 0.1 mol L^−1^ HCl and followed with distilled water for several times by centrifugation until pH neutral was obtained. BKP with or without enzymatic pretreatment was diluted to a solid concentration of 0.1% (w/v) and nanofibrillated using a sonicator (Fisher Model 500) at 30% amplitude (150 W) for 20 min. The nanofibrillated BKP was indicated as NBKP and stored in a refrigerator at 4 °C for further analysis.

### Substrates physiochemical characteristics

Released sugars in the liquid fraction were determined by HPLC analysis as previously described. Briefly, 0.7 mL of 72% H_2_SO_4_ was added to 15 mL of the liquid samples and the volume was adjusted to 20 mL with water. Then the samples were autoclaved at 121 °C for 1 h before being analyzed by HPLC.

X-ray Diffraction patterns (XRD) of BKP with or without enzymatic pretreatment were collected on a Bruker D8-Advance powder x-ray diffractometer, using Cu-Ka radiation (k = 0.1540 nm) at an accelerating voltage of 40 kV and a current of 40 mA. The data were collected from 2θ = 5–80° with a step interval of 0.04°. The percentage of crystallinity index (% CrI) was calculated by Segal method according to Eq. 3^32^:

1 $$CrI( \% )=\frac{{I}_{002}-{I}_{am}}{{I}_{002}}\times 100$$

where I_002_ is the maximum intensity of around 22.5° and I_am_ corresponds to the minimum intensity located at 2θ close to 18°. The average crystal size τ was determined by the Scherrer equation (Eq. 2)^33^,

2 $$\tau =\frac{K\lambda }{\beta \,\cos \,\theta }$$

where K is a constant that depends on the crystal shape (1.0 in this case), λ is the wavelength of the incident beam in the diffraction experiment, β is the full width at half maximum in radians and θ is the position of the peak (half of the plotted 2θ value).

The average degree of polymerization (DP) was calculated based on the intrinsic viscosity value by Eq. 3^34^,

3 $$D{P}^{0.905}=0.75[\eta ]$$

where [η] is intrinsic viscosity in cm^3^ g^−1^ determined by viscosity (25 °C) of cellulose solution in cupri ethylenediamine (CED) solution using Ubbelohde viscometer according to ASTM D1795.

FTIR spectra were recorded on a spectrophotometer (Perkin Elmer, Wellesley, MA) equipped with a ZnSe window by averaging 32 scans from 4000 to 400 cm^−1^ at 4 cm^−1^ resolution. All the cellulose FTIR spectra were normalized at 1060 cm^−1^ (–C–O– stretching vibration of glucose ring,) to make a fare compare among those FTIR spectra (a common FTIR processing method for cellulose).

The width and length of BKP after enzymatic pretreatment was monitored on a fiber quality analyzer (FQA, LDA02; OpTest Equipment, Inc., Hawkesbury, ON, Canada) according to the procedure described previously^25^.

The water retention value (WRV) was determined and calculated according to TAPPI Useful Method-256. Briefly, 0.5 g (oven dry weight) of never dried pulp was suspended in 50 ml deionized water and shaken vigorously to break the pulp apart. The pulp slurry was allowed to soak overnight at room temperature and then centrifuged (900 G, 25 °C) for 30 minutes. The wet pulp pad after centrifugation were then weighted and dried overnight at 105 °C oven and reweight. WRV was calculated as the weight of water retained in the pulp pad after centrifugation divided by the dry weight of the fibres according to the following equation (Eq. 4),

4 $$WRV=\frac{(Ww-Wd)}{Wd}\,$$

where W_w_ is the weight of the wet sample after centrifuging, and W_d_ is that of the dried pulp.

The carboxylic acid group content of the pretreated BKP was measured using the conductometric method according to previous reports^35^. Zeta potential of NBKP was measured using Zetasizer nano-ZS (Malvern, CA).

The visible light transmittance of sonicated NBKP fiber suspension (0.1%, w/v) was measured at 600 nm on a Cary® 50 UV-Vis spectrophotometer.

Observations of BKP and NBKP fibers were performed using a scanning electron microscopy (SEM) (Hitachi S3000N VP-SEM) equipment. A thin layer of Pd–Au alloy was coated on the specimen prior to measurement to prevent charging on the surface. The diameters of the NBKP fiber from SEM images were quantified using the imaging software Image J. About 100 fibers were randomly and manually picked for analysis.

Thermogravimetric analysis (TGA) of the obtained NBKP samples was measured on a TG209 F1 instrument (NETZSCH Co., Germany). About 5–8 mg of each sample was heated in a platinum crucible from room temperature to 600 °C at a heating rate of 20 °C min^−1^ under nitrogen atmosphere.

All of the experiments were performed in triplicate and mean values and errors bars were calculated and reported. The statistical analysis showed that the difference were statistically significant (one way ANOVA, p < 0.05).

## Electronic supplementary material

Supplementary Information
