# Supplementary material for: Enzyme mediated nanofibrillation of cellulose by the synergistic actions of an endoglucanase, lytic polysaccharide monooxygenase (LPMO) and xylanase
Source: Sci Rep. 2018 Feb 16;8:3195. doi: 10.1038/s41598-018-21016-6 (PMC5816652; doi:10.1038/s41598-018-21016-6)
Supplement: Supplementary file 1 — Supplementary Information [file 41598_2018_21016_MOESM1_ESM.pdf]

Enzyme mediated nanofibrillation of cellulose by the synergistic actions of an endoglucanase, lytic polysaccharide monooxygenase (LPMO) and xylanase

Jinguang Hu[1], Dong Tian[1,3], Scott Rennecker[2], and Jack N. Saddler[1*]

1. Forest Products Biotechnology/Bioenergy Group, Department of Wood Science, Faculty of Forestry, University of British Columbia,2424 Main Mall, Vancouver, British Columbia V6T 1Z4, Canada
2. Advanced Renewable Materials Lab, Department of Wood Science, Faculty of Forestry, University of British Columbia,2424 Main Mall, Vancouver, British Columbia V6T 1Z4, Canada
3. Institute of Ecological and Environmental Sciences, Sichuan Agricultural University, Chengdu, Sichuan 611130, PR China

**Corresponding author: Jack N. Saddler (jack.saddler@ubc.ca)*

Supplementary Information

Table S1. The gross fiber properties (fiber length and fiber width) of bleached Kraft pulp (BKP) before and after 3 hours treatment with various enzyme.

Sample	Fiber length (μm)	Fiber width (μm)
Control	761.3 ± 28.9	21.0 ± 0.3
EG	765.1 ± 15.3	20.8 ± 0.8
EG + AA9	753.8 ± 23.3	20.7 ± 1.0
EG + EX	748.6 ± 31.2	20.5 ± 0.3
EG + EX + AA9	746.9 ± 30.8	20.6 ± 0.5

EG: endoglucanase; AA9: lytic polysaccharide monooxygenase auxiliary activity family 9 enzyme; EX: endoxylanase. The experiment were performed in triplicate and the mean values and errors bars were calculated.

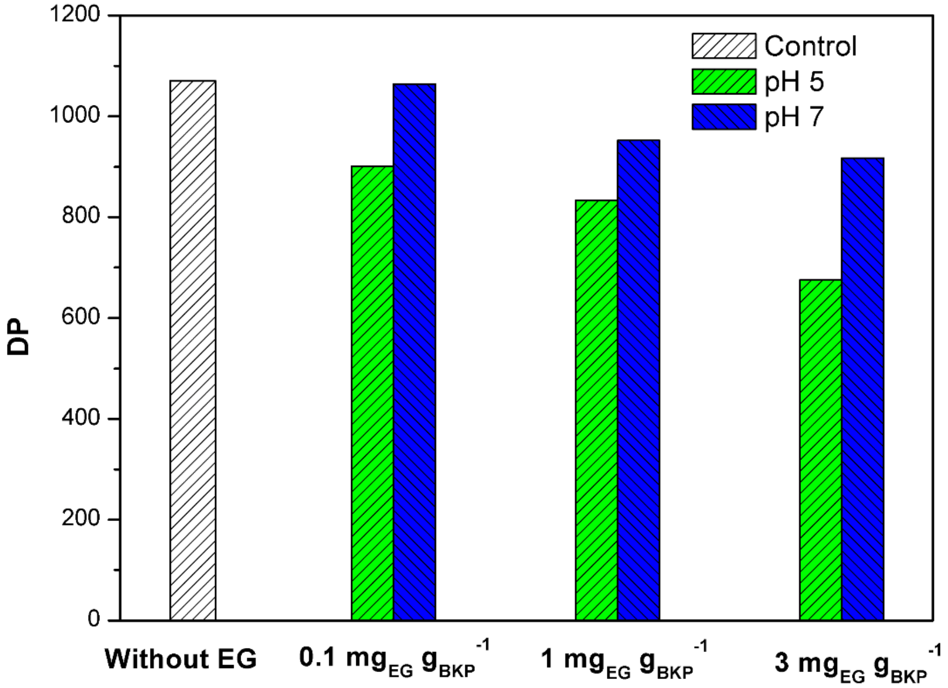

Fig. S1 Reduction in the degree of polymerization (DP) of bleached Kraft pulp (BKP) after 3 hours endoglucanase (EG) treatment at different enzyme loadings at pH 5.0 and 7.0.

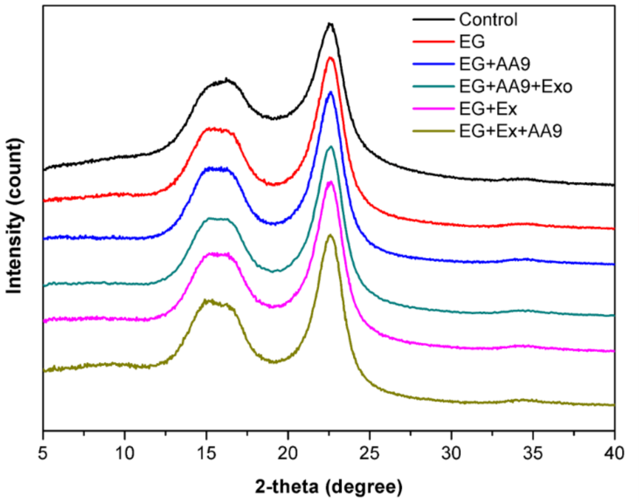

Sample	Segal CrI (%)	Crystal size (nm)
Control	53.5	2.5
EG	60.8	3.8
EG + AA9	63.2	4.0
EG + AA9 + Exo	59.7	3.8
EG + EX + AA9	71.1	3.9
EG + EX	66.0	3.8

Fig. S2 Changes in fiber crystallinity index (CrI) and crystal size (τ) of bleached Kraft pulp (BKP) after various enzyme treatments.

EG: endoglucanase; AA9: lytic polysaccharide monooxygenase auxiliary activity family 9 enzyme; EX: endoxylanase; Exo: exoglucanase Cel6A.

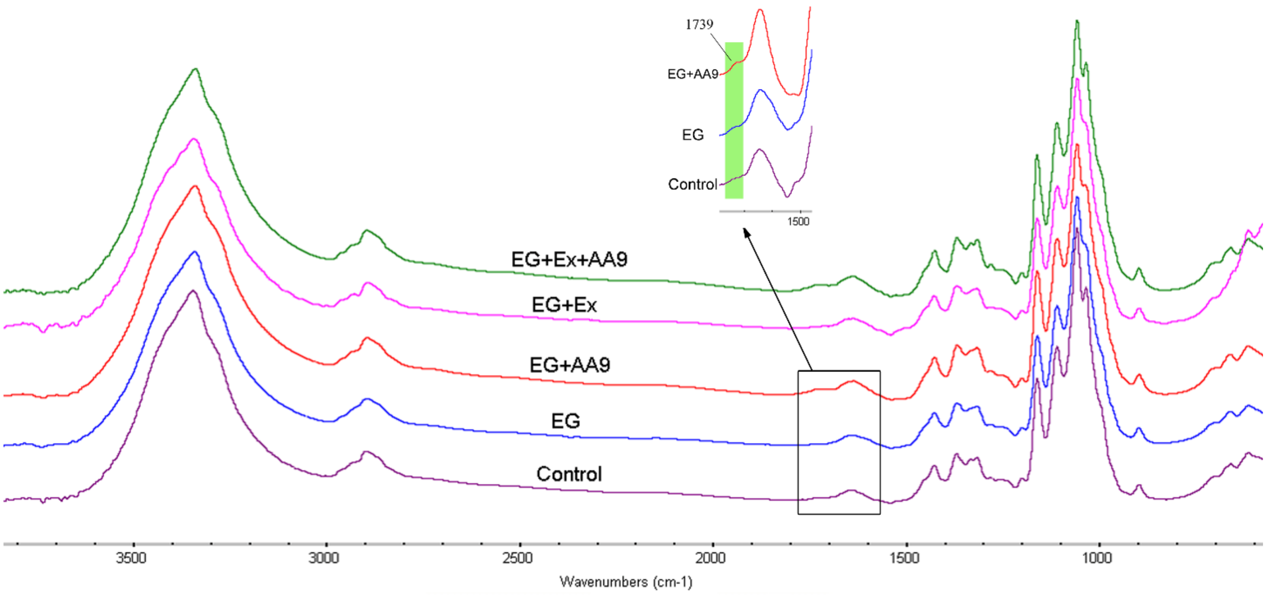

Fig. S3 FT-IR spectrometry of bleached Kraft pulp (BKP) treated by various enzyme combinations.

EG: endoglucanase; AA9: lytic polysaccharide monooxygenase auxiliary activity family 9 enzyme; EX: endoxylanase.

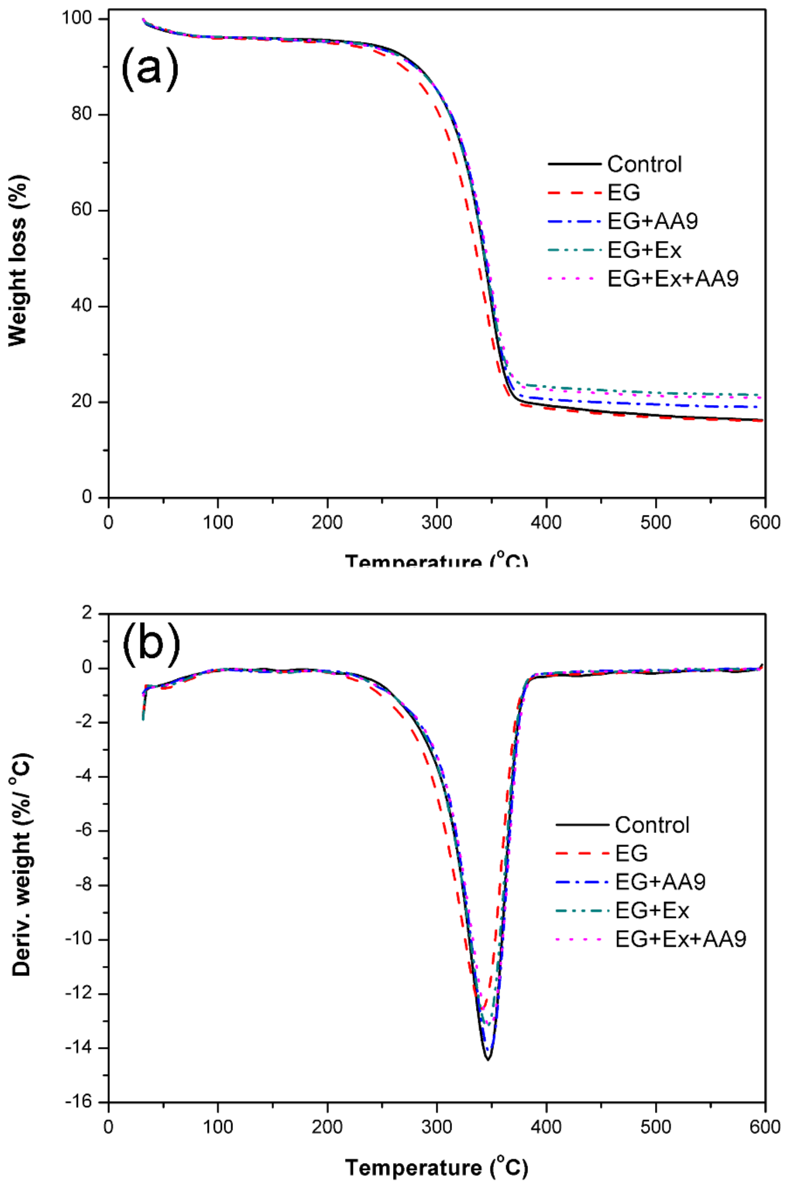

Fig. S4 TGA (a) and DTG (b) curves of enzymatically pretreated bleached Kraft pulp (BKP) after sonication and assessed by thermal gravimetric analysis (TGA).

EG: endoglucanase; AA9: lytic polysaccharide monooxygenase auxiliary activity family 9 enzyme; EX: endoxylanase.

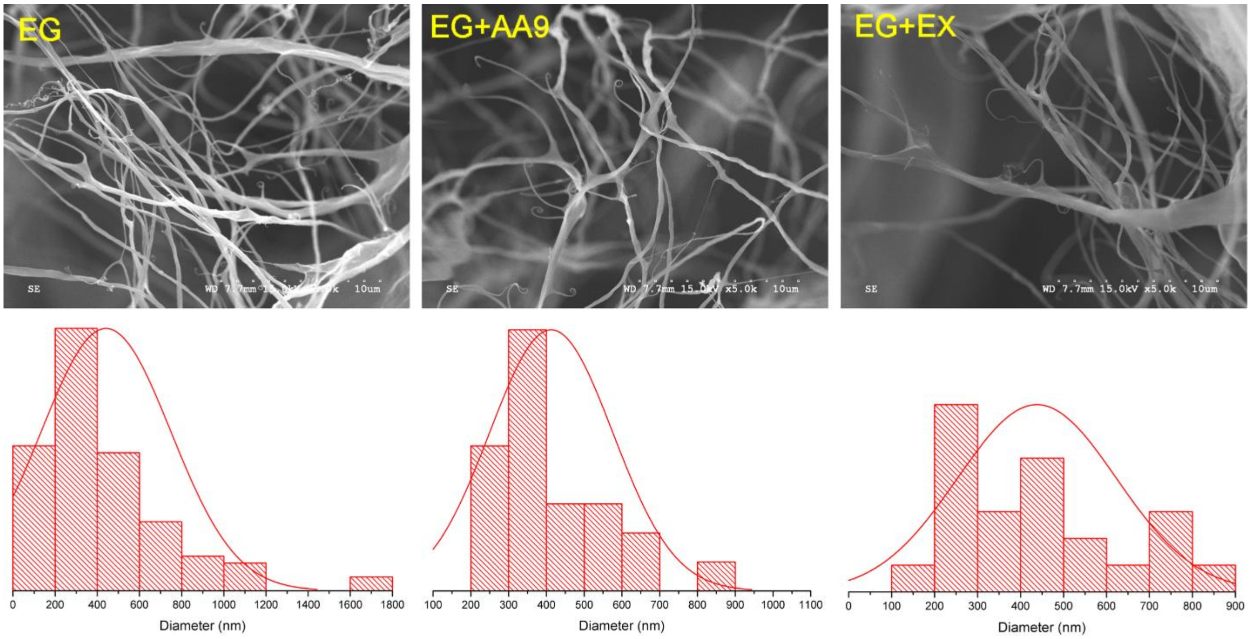

Figure S5. SEM images of enzymatically pretreated bleached kraft pulp (BKP) fiber suspension after sonication process (A) and the fiber width distribution of pretreated sample (B).

EG: endoglucanase; AA9: lytic polysaccharide monooxygenase auxiliary activity family 9 enzyme; EX: endoxylanase.
